# High-cost users: drivers of inpatient healthcare expenditure concentration in urban China

**DOI:** 10.1186/s12913-022-08775-9

**Published:** 2022-11-14

**Authors:** Qiuyan Fan, Jian Wang, Stephen Nicholas, Elizabeth Maitland

**Affiliations:** 1 Dong Fureng Institute of Economic and Social Development, Wuhan University, Wuhan, 430072 China; 2 Australian National Institute of Management and Commerce, Eveleigh, Sydney, NSW 2015 Australia; 3 School of Economics and School of Management, Tianjin Normal University, Tianjin, 300074 China; 4 Newcastle Business School, University of Newcastle, Newcastle, NSW 2308 Australia; 5 Research Institute for International Strategies, Guangdong University of Foreign Studies, Guangzhou, 510420 China; 6 School of Management, University of Liverpool, Chatham Building, Chatham Street, Liverpool, L697ZH England

**Keywords:** Healthcare expenditures, Concentration, High-cost users, Recentered influence functions

## Abstract

**Background:**

Total healthcare expenditures are concentrated among a small number of patients. To date, studies on the concentration of health care expenditures in developing countries are limited, mainly focusing on concentration measures and the demographic, clinical and socioeconomic characteristics of high-cost users (HCU). The drivers of the skewed overall distribution of health care expenditures are opaque. Using inpatient administrative claims data, this study provides new evidence on the concentration of healthcare expenditures in China; the demographic and clinical characteristics of high-cost users; and the drivers of the overall distribution of healthcare expenditures.

**Methods:**

Utilizing administrative claims data for hospitalization in a prefecture-level city in China, we investigated the concentration of healthcare expenditure. We used recentered influence function (RIF) regression to examine the drivers of healthcare expenditure concentration, decomposing and estimating the effects of demographic and disease characteristics on the overall distribution of health care expenditures.

**Results:**

Using a sample of 87,841 adults, we found extreme skewness in the distribution of inpatient medical expenditures in China, with approximately 49% of annual medical expenditures generated by the top 10% of inpatient groups. HCUs tend to be elderly and male, with high-frequency hospitalizations and long lengths of stay. In addition, healthcare expenditure concentration was related to diseases of the circulatory system, malignant neoplasms, diseases of the musculoskeletal system and connective tissue, diseases of the digestive system, injury and poisoning, and diseases of the respiratory system. Malignant and major diseases reinforced the concentration of healthcare spending, and a 10% increase in the prevalence of malignancy would result in a predicted Gini coefficient increase of 7.2%, heart disease of 0.92% and cerebrovascular disease of 1.5%. The above significant positive effects were not observed for hypertension and diabetes mellitus.

**Conclusions:**

Our study provides new insights into the concentration of inpatient medical expenditures in China, including the precise picture of HCU expenditure concentration, the drivers of HCU expenditure concentration and the magnitude of their impact. With the aging of China's population and the profound shift in the disease spectrum, policymakers need to strengthen the early detection and intervention management of specific chronic diseases and high-risk populations, especially the early diagnosis and treatment of key cancers.

## Introduction

The concentration of a disproportionate share of healthcare expenditures on a small proportion of patients, or high-cost users (HCU), is typical of healthcare expenditures everywhere. While this concentration, or skewness of health care expenditures, has been extensively documented [2, 28, 32], the evidence of medical spending concentration has mainly focused on developed countries. Empirical evidence from the United States revealed that the top 5% of healthcare spenders accounted for around 50% of total health care expenditures [1, 5, 15] and France and Japan showed a similar concentration, with the top 10% of patients accounting for more than 60% of all health care spending [13, 16]. Due to the lack of publicly available healthcare expenditure data and reliable large sample survey data, only a limited number of published studies have been carried out in developing countries. Using insurance claims data, Chen et al. [3] and Peng and Du [23] suggested that nearly 50% of inpatient medical expenditures in China were generated by the top 10% of patients. The concentration of outpatient expenditures in Iran was even higher, with the top 10% of patients accounting for 63% of the total ambulatory expenditure [8].

Given that a small subset of the patient population imposes a disproportionately high-cost burden on the health care system, one focus of the healthcare concentration expenditure literature has been on the assessment of the concentration, identifying and understanding the characteristics of these HCUs. HCUs are typically older, experience frequent hospitalizations, have lower incomes [14] and suffer multiple chronic common medical conditions, including cardiovascular disease, cancer, chronic obstructive pulmonary disease and diabetes [8, 17, 21].

Although extensive research has been conducted on the concentration of health care spending, what is less clear is the drivers of the skewed distribution of health care spending, particularly demographic characteristics and the disease profile. Utilizing patient claims data, we address three questions. First, we statistically describe the distribution of urban inpatient medical expenditures. Second, we identify the demographic and disease characteristics of HCUs that differentiate them from the other healthcare users. Third, we assess the underlying drivers of concentrated healthcare expenditures, such as the demographic and disease characteristics.

## Data and method

### Data

We were given permission to use anonymized cross-sectional administrative claims data on hospitalization expenditures by Urban Employee Basic Medical Insurance (UEBMI) insured patients in a prefecture-level central China city in 2020. The condition on the use of the UEBMI data was that the city could not be named, so our results are generalizable to central regions of China, but not the whole country. UEBMI is a compulsory scheme for employed and retired urban workers with premiums paid by the employer and the employee. Administrative claims data provides detailed individual-specific information, such as an anonymized identification number, hospital visited, disease diagnosis, length of hospital stay, and total medical expenditures, generated by the hospital information system and health insurance information system. It is important to note that personally identifiable information, including patient name and health insurance card number, was anonymized, but each patient was given a unique individual-specific patient ID. This anonymous patient-specific ID is important since 28% of individuals were hospitalized multiple times during 2020. Cost and utilization data from all visits within the calendar year were aggregated and reported for each patient, yielding data on 87,841 inpatient medical expenditure records.

### Method

#### Statistical analysis

To describe the concentration of medical expenditures, we first calculated the means of different quantiles of annual inpatient medical expenditures by age and sex, and then compared the differences in medical expenditures across quartiles. We also calculated the Gini coefficient, a measure of inequality, for medical expenditures, the proportion of HCUs (top 5% and top 10%) in total expenditures, and Lorenz curves to visualize the concentration of medical expenditure distribution. In addition, to understand the characteristics of high-cost users, we examined the statistical differences between high-cost users and the other user groups by between-group difference tests. Finally, we relied on disease codes to classify the main diseases and to create the distribution characteristics of high-cost users across disease types.

#### Recentered influence regression

Concentration portrays the overall distribution characteristics, which can be measured by indicators such as Gini coefficient, quantile distance, and variance. To examine the effect of the drivers of the overall healthcare expenditure distribution, the usual regressions models are unsuitable. The problem is that the healthcare expenditure concentration, as a distributional characteristic, is fixed under a given sample and without variability. While general regression strategies, such as ordinary least squares, can estimate the effects of factors on individual health care expenditures, standard regression models cannot directly capture the drivers of the overall healthcare expenditure distribution with constant distribution characteristics. To address this problem, we use the unconditional quantile regression [11] to explore the effect of the drivers of the concentration of health care expenditures. The key concept in the unconditional quantile regression is the influence functions (IFs), which are used to analyze the robustness of the distributional statistics to small disturbances in the data [6]. Firpo et al. [11] suggested using recentered influence functions (RIFs) to estimate the small changes in the distribution of the independent variables on the distributional measure of interest, such as Gini coefficient, quantile, and variance.

### Variables

#### Dependent variable

The dependent variable was each UEBMI patient’s total inpatient healthcare expenditure for the calendar year 2020.

#### Independent variables

The HIS and insurance information system contained demographic variables, including age and sex, while inpatient claims records provided definitive diagnosis information and corresponding classification and codes of diseases (CCD). The CCD is compiled from the Chinese classification and codes of disease (GBT 14,396–2016), which is an extension of international classification of diseases (ICD-10) and is China’s authoritative standard for disease classification. We calculated the inpatient length of stay (LOS), frequency of hospitalizations and total number of disease categories during 2020 based on admission and discharge dates, where 28% of patients had multiple hospital stays. In addition, the hospital level was classified into primary, secondary and tertiary hospitals.

## Results

### Statistical analysis

**Fig. 1:**
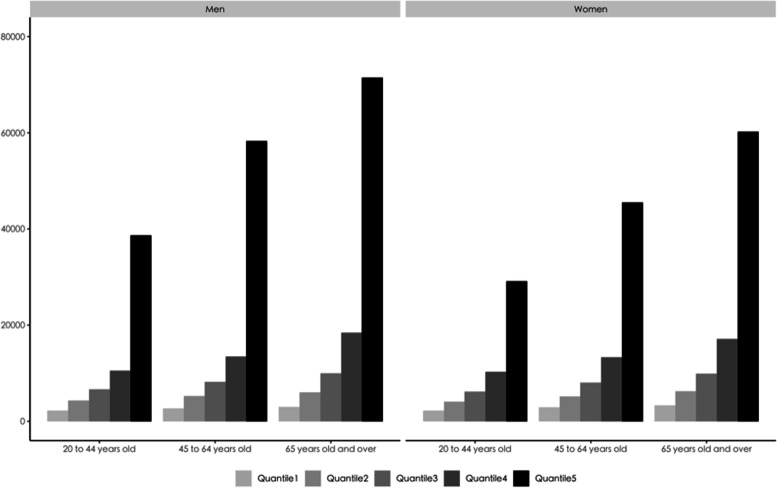
Average total medical expenditure, by age, sex and expenditure quintile

Figure 1 displays average hospital expenditures by quintiles, disaggregated by sex and age groups. In terms of quartile means, the average spending of the fifth quartile were between 13 times greater for females and 23 times greater for males than that of the first quartile. Inpatient expenditures increased with age driving the concentration of expenditures. The ratio of average expenditures for men in the top quintile to those in the bottom quintile increased from 17.2 times for those aged 20–44 to 21.4 times for those aged 45–64, reaching a peak of 23.9 times at age 65 and older.

**Fig. 2:**
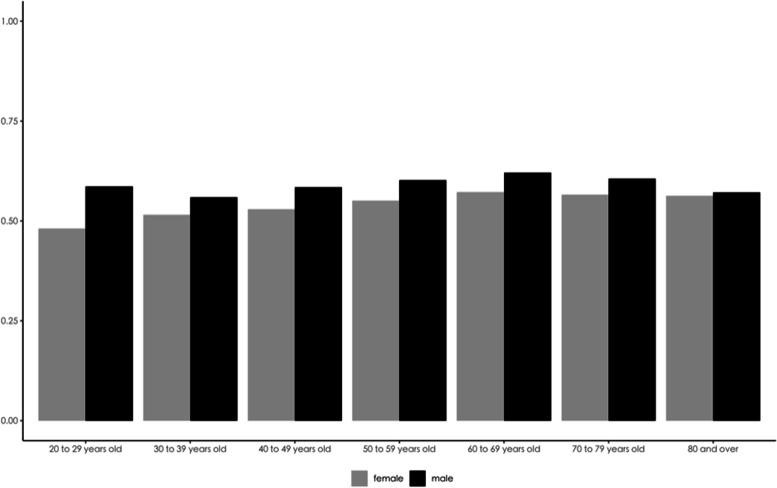
Sex-age differences in inpatient expenditure concentration

In Fig. 2, we further examined the sex differences in the concentration of healthcare expenditures by age group through the Gini coefficient. Figure 2 shows that the healthcare expenditure concentration was greater for men than for women in all age groups, but the gap decreased with age. Specifically, the difference in the concentration between men and women peaked at 0.106 for 20 to 29 years old group and then continuously decreased. At age 80 and older, the difference between males and females declined to 0.009, with almost no difference.

**Fig. 3:**
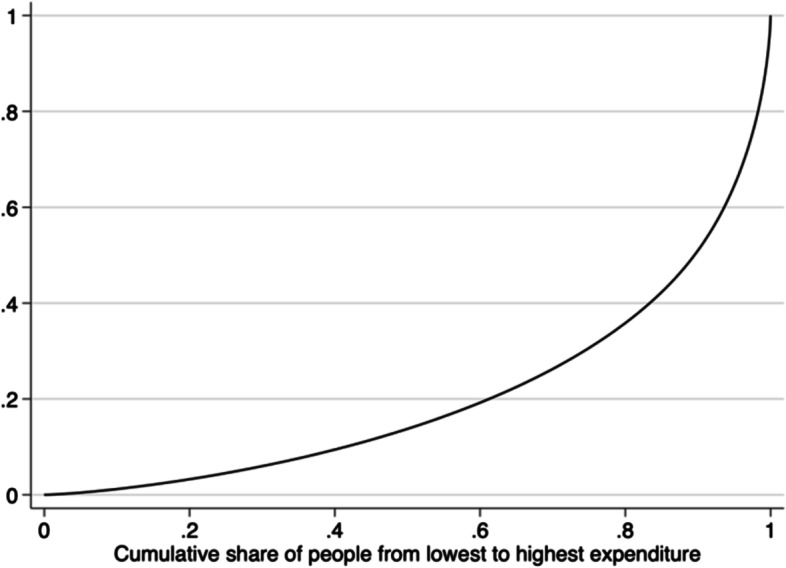
Lorenz concentration curve of medical expenditure

The Gini coefficient was 0.58, indicating a very high HCU concentration of inpatient medical expenditures. As shown in the Lorenz concentration curve in Fig. 3, the top 10% of inpatients accounted for 49% of overall inpatient expenditures and the top 20% accounted for 73.8% of inpatient expenditures. In terms of all UEBMI members, 1.67% (0.8786 / 52.5) of the total subscribers consumed close to 50% of the annual inpatient medical expenditures. The above results confirm that impatient healthcare expenditure was disproportionately concentrated in a small subset of the insured.

### Demographic and disease characteristics of high-cost users

Given the skewness of healthcare expenditure, understanding the characteristics of HCUs is of great practical importance to control overall healthcare cost growth. We estimated the statistical differences between HCUs and other groups in terms of their demographic characteristics, disease classification, and health care utilization. The percentage threshold used to identify HCUs varied across different studies, ranging from top 5% [12, 25, 28] to top 20% [23], with top 10% the most commonly used threshold for HCU identification [17, 18, 28]. We considered the HCUs to be patients in the top 5% and the top 10% of total medical expenditure. As shown in Table 1, high-cost users were older and more likely to be male.

**Table 1:** Demographic and disease characteristics of high-cost users

Variables	The bottom 95%	The top 5%	Difference	The bottom 90%	The top 10%	Difference
*Dependent variable*						
Medical expenditure	11,512.925	120,346.312	108,833.391^***^	9602.975	83,119.82	73,516.844^***^
	(10,864.594)	(89,652.766)	(1353.181)	-7243.367	-73,686.344	(786.600)
*Demographic*						
Age	57.412	63.737	6.325^***^	57.112	63.269	6.157^***^
	(14.496)	(12.598)	(0.197)	(14.491)	(13.067)	(0.149)
Sex	0.520	0.647	0.127^***^	0.516	0.622	0.106^***^
	(0.500)	(0.478)	(0.007)	(0.500)	(0.485)	(0.005)
*Disease diagnosis*						
Malignant tumor	0.018	0.296	0.278^***^	0.012	0.212	0.200^***^
	(0.133)	(0.457)	(0.007)	(0.109)	(0.409)	(0.004)
Heart disease	0.087	0.228	0.140^***^	0.080	0.229	0.150^***^
	(0.283)	(0.419)	(0.006)	(0.271)	(0.420)	(0.005)
Cerebrovascular disease	0.065	0.150	0.085^***^	0.062	0.130	0.068^***^
	(0.246)	(0.357)	(0.005)	(0.242)	(0.336)	(0.004)
Hypertension	0.045	0.036	-0.009^***^	0.046	0.036	-0.010^***^
	(0.208)	(0.186)	(0.003)	(0.209)	(0.185)	(0.002)
Diabetes	0.041	0.039	-0.002	0.041	0.037	-0.004^*^
	(0.198)	(0.193)	(0.003)	(0.199)	(0.189)	(0.002)
Injury, poisoning	0.037	0.066	0.029^***^	0.034	0.077	0.042^***^
	(0.189)	(0.249)	(0.004)	(0.182)	(0.266)	(0.003)
Disease category number	1.304	2.753	1.448^***^	1.259	2.435	1.176^***^
	(0.655)	(1.592)	(0.024)	(0.577)	(1.447)	(0.016)
*Medical service utilization*						
Number of hospitalizations	1.379	3.841	2.462^***^	1.314	3.187	1.873^***^
	(0.814)	(2.898)	(0.044)	(0.680)	(2.459)	(0.026)
Inpatient length of stay	14.739	61.923	47.184^***^	13.661	48.034	34.373^***^
	(14.438)	(57.366)	(0.867)	(12.106)	(47.968)	(0.514)
Hospital level	2.322	2.748	0.426^***^	2.302	2.717	0.414^***^
	(0.730)	(0.490)	(0.008)	(0.733)	(0.523)	(0.006)
Observations	83,449	4392	87,841	79,057	8784	87,841

Robust errors in parentheses

^***^ *p* < 0.01, ** *p* < 0.05, * *p* < 0.1

In terms of disease profiles, the HCUs in Table 1 had a higher prevalence of chronic diseases, such as malignant tumors, heart disease, and cerebrovascular disease; lower prevalence of hypertension; and no significant differences for diabetes compared with the other groups. Our disease categories were derived from inpatient claims records, but hypertension and diabetes in China were largely treated in outpatient clinics [19]. In addition, Table 1 reveals that the number of hospitalizations, length of stay, level of hospital and number of hospitalized diseases were significantly higher in HCUs, reflecting the frequency of hospitalization and coexistence of multiple diseases.

The concentration of health costs is impacted by the distribution of the disease burden [14]. Inpatient claims records reported detailed diagnosis codes, which allowed us to further analyze the distribution of primary diseases among HCUs. As shown in Table 2, the top 6 disease categories in the top 10% healthcare expenditure group accounted for 71.7% of the disease types, comprising the circulatory system (29.07%), tumors (17.31%), diseases of the musculoskeletal system and connective tissue (7.82%), diseases of the digestive system (5.9%), injuries, poisoning and certain other consequences of external causes (5.53%), and diseases of the respiratory system (5.51%). Diseases of the circulatory system were dominated by heart disease and cerebrovascular disease, which together accounted for 79.09% of circulatory system diseases and heart disease 14.3% and cerebrovascular disease 8.7% of all cases. Among the tumors, malignant tumors were predominant, with malignant tumors of digestive organs accounting for 4.77%, and respiratory and intrathoracic organs accounting for 4.05%, of all cases. Diseases of musculoskeletal system and connective tissue had a high prevalence of back disorders, spinal disorders, and joint disorders. Diseases of digestive system were dominated by gallbladder, biliary and pancreatic disorders, liver diseases, esophageal, gastric and duodenal diseases. Injury, poisoning and certain other consequences of external causes accounted for most of hip and thigh injuries, knee and calf injuries, and shoulder and upper arm injuries, which together accounted for 3.53% of the total cases. These disease results show that the categories of diseases in the highest 10% of inpatient medical expenditures were characterized by chronicity and severity, with malignant tumors, cerebrovascular diseases, and heart diseases occupying the highest proportion.

**Table 2:** Distribution of primary diagnoses in high-cost users

Disease classification	observations	Ratio (%)	
		Total	Inner Category
Diseases of the circulatory system	2554	29.07	
Ischemic heart disease	1256	14.30	49.18
Cerebrovascular disease	764	8.70	29.91
Other forms of heart disease	278	3.16	10.88
Others	256	2.91	10.02
Tumors	1521	17.31	
Malignant tumors of digestive organs	419	4.77	27.55
Malignant tumors of respiratory and intrathoracic organs	356	4.05	23.41
Benign tumors and tumor in situ	86	0.98	5.65
Malignant tumors of lymph, hematopoietic and related tissues	71	0.81	4.67
Malignant tumors of breast	64	0.73	4.21
Other malignant tumors	319	3.63	20.97
Diseases of the musculoskeletal system and connective tissue	687	7.82	
Other dorsopathies	255	2.90	37.12
Spondylopathies	112	1.27	16.30
Arthropathies	57	0.65	8.30
Others	263	2.99	38.28
Diseases of the digestive system	518	5.90	
Disorders of gallbladder, biliary tract and pancreas	186	2.12	35.91
Diseases of liver	117	1.33	22.59
Diseases of oesophagus, stomach and duodenum	64	0.73	12.36
Others	151	1.72	29.15
Injury, poisoning and certain other consequences of external causes	486	5.53	
Injuries to the hip and thigh	178	2.03	36.63
Injuries to the knee and lower leg	75	0.85	15.43
Injuries to the shoulder and upper arm	57	0.65	11.73
Others	176	2.00	36.21
Diseases of the respiratory system	484	5.51	
Chronic lower respiratory diseases	167	1.90	34.50
Influenza and pneumonia	120	1.37	24.79
Other diseases of the respiratory system	113	1.29	23.35
Others	84	0.96	17.36
Total	6250	71.14	

### Drivers of high-user healthcare expenditure concentration

Understanding the drivers of the underlying causes of healthcare expenditure concentration variation has implications for medical cost control. While we provided a statistical description and characterization of concentration, we need to directly identify the direction and magnitude of the underlying causes of healthcare expenditure concentration across the entire distribution. To address this challenge, we carried out quantile decomposition analyses to quantify the magnitude of the drivers on the changes in the overall distribution. Following the general approach of the healthcare literature [24], we used the Gini coefficient, the difference between the 90th and 10th quartiles, and the variance to depict the drivers of the concentration of medical expenditures.

Table 3 shows the results of RIF regressions for selected distributional statistics. For different concentration measures, all the models produced consistent results with different insights. A higher concentration of medical expenditures resulted from an increase in the proportion of males, the first driver of healthcare expenditure concentration. Specifically, a 10% increase in the overall proportion of males would increase the Gini coefficient by 0.64% (0.038/0.588 × 0.1), the log hospitalized costs gap between the 90th and 10th would increase 2.46% and variance of log hospitalized costs would increase 1.4% (0.146/1.013 × 0.1).

**Table 3:** Drivers for inpatient expenditures concentration

	(1)	(2)	(3)
	Gini	Quantile (90 10)	Variance
Sex	0.038^***^	0.246^***^	0.146^***^
	(0.00)	(0.02)	(0.01)
Malignant neoplasms	0.421^***^	2.914^***^	1.720^***^
	(0.03)	(0.09)	(0.05)
Heart disease	0.054^***^	0.858^***^	0.347^***^
	(0.01)	(0.04)	(0.02)
Cerebrovascular disease	0.088^***^	0.208^***^	0.223^***^
	(0.01)	(0.04)	(0.03)
Hypertension	-0.002	-0.152^***^	-0.089^***^
	(0.01)	(0.04)	(0.02)
Diabetes	-0.061^***^	-0.454^***^	-0.321^***^
	(0.01)	(0.04)	(0.02)
Injury and poisoning	0.023^*^	0.746^***^	0.341^***^
	(0.01)	(0.05)	(0.03)
Length of stay	0.014^***^	0.054^***^	0.041^***^
	(0.00)	(0.00)	(0.00)
Disease category number	-0.070^***^	0.164^***^	0.041^***^
	(0.01)	(0.02)	(0.01)
Tertiary hospital	-0.060^***^	0.021	-0.070^***^
	(0.00)	(0.02)	(0.01)
Constant	0.441^***^	1.068^***^	0.144^***^
	(0.01)	(0.04)	(0.02)
Age fixed effect	Yes	Yes	Yes
Average RIF	0.588	2.515	1.013
adj. *R*^2^	0.134	0.209	0.232
*N*	87,841	87,841	87,841

Bootstrap standard errors in parentheses based on 500 repetitions. All models included age fixed effects

^***^ *p* < 0.01, ** *p* < 0.05, * *p* < 0.1

Chronic and malignant diseases have been identified as key drivers of the concentration of inpatient medical expenditures [2, 10], but the actual effect remains ambiguous. Our models incorporated binary variables for disease diagnosis. We selected malignancy, heart disease, and cerebrovascular disease as the most prevalent diseases accounting for high-cost users’ hospitalization and the top cause of death in China [20, 31]. In addition, hypertension and diabetes were included as prevalent chronic diseases in China [27]. The direction and magnitude of different diseases on the concentration of inpatient expenditures differed. If the proportion of hospitalized patients with malignant neoplasms increased by 10%, from 3.2% as currently observed to 13.2%, the predicted Gini coefficient would increase by 7.2% (0.421/0.588 × 0.1), the log hospitalized costs gap between the 90th and 10th would increase 29%, and the variance of log costs would increase by 17.0% (1.720/1.013 × 0.1). This suggested that the low prevalence of serious diseases, represented by malignant neoplasms, but accompanied by high expenditure characteristics, was an important driver of healthcare expenditure concentration. Heart disease and cerebrovascular disease had similar positive effects on concentration as malignant neoplasms, but to a lesser extent. Taking the Gini coefficient as an example, the predicted Gini coefficient would increase by 0.92 percent (0.054/0.588 × 0.1) for a 10 percent increase in the overall prevalence of heart disease and 1.5 percent (0.088/0.588 × 0.1) for cerebrovascular disease. Hypertension and diabetes, which are common chronic diseases, did not significantly increase the concentration. Using the Gini coefficient as an example, a 10% increase in the overall proportion of diabetes would decrease the predicted Gini coefficient by 1.0 percent (-0.061/0.588 × 0.1), and hypertension reduced the Gini coefficient of inpatient expenditures, but the change was not statistically significant. A 10% increase in the proportion of injury and poisoning, the predicted Gini coefficient would increase only 0.39 percent (0.023/0.588 × 0.1) at the 10% significant level.

In terms of inpatient service utilization in Table 3, if the average inpatient length of stay increased by 1 day, the predicted Gini coefficient would rise by 2.3 percent (0.014/0.588), the log hospitalized costs gap between the 90th and 10th percentiles could increase by 3.8 percent, and the variance of log hospitalized costs would increase by 14.4 percent (0.146/1.013). Unlike the effect of inpatient length of stay, type of hospital in Table 3 did not significantly increase expenditure concentration. The predicted Gini coefficient would decrease by 1.0 percent (-0.060/0.588 × 0.1) and the variance of log hospitalized costs would decrease by 0.69 percent (-0.070/1.013 × 0.1) with a 10% increase in the proportion of the overall patient population choosing tertiary institutions.

## Discussion

Little is known about the drivers of healthcare expenditure concentration in developing countries and especially in China. Using insurance claims data, we examined the concentration of healthcare expenditures and the drivers of urban inpatient healthcare expenditures. Our results revealed that Chinese healthcare expenditures were highly concentrated in a small subset of the insured, similar to empirical evidence from developed countries [1, 5, 13, 15, 16]. The top 10% of hospitalized groups accounted for approximately 49% of annual medical expenditures, or only 1.67% of the UEBMI insured consumed close to 50% of annual inpatient medical expenditures. This is generally consistent with the findings of Peng and Du [23] and Chen et al. [3] regarding the concentration of inpatient medical expenditures in China. Despite some evidence suggesting that concentration decreased with age [22] and varied by sex [13], our results for the ratio of average expenditure in the quintile revealed that concentration increased with age for both males and females. Sex differences existed in the concentration of inpatient medical expenditures, with males having a greater concentration than females in the same age group at all ages, but this difference decreased with age, disappearing by age 80.

One major contribution of our study was to deconstruct the drivers of high-cost user concentration of healthcare spending, by exploring the demographic and clinical characteristics of HCUs. Given the limitation of the insurance claim data to age and sex demographics [12], we first compared age-sex factors between distinct healthcare expenditure subgroups. Our results showed that HCUs were older and male, which is consistent with previous research on high-cost users [25] and explains the increase in the concentration of inpatient medical expenditures with population aging. In terms of disease profiles, it has been well documented that the high burden of comorbid diseases, especially multiple chronic status, is a major driver of health care spending [2, 25]. Our empirical evidence from China showed that the incidence of chronic diseases such as malignancy, heart disease, and cerebrovascular disease was significantly higher in HCUs than in non-HCUs and was often accompanied by overlapping chronic diseases. Our results also showed that the coexistence of multiple diseases increased the intensity of health care utilization among HCUs, resulting in higher number and length of hospital stays.

Our study is novel in that we quantify the drivers for inpatient expenditures concentration. Previous studies have confirmed the basic axiom that health care expenditures were concentrated in specific sex-age subgroups [15, 23] and identified a range of diseases that elevated the probability of being a HCUs [6, 14, 17]. However, with only qualitative assumptions [23], the impact of potential drivers on changes in concentration remains ambiguous, and a rigorous quantitative assessment lacking. To bridge this research gap, we examined the drivers of inpatient medical expenditure concentration using the recentered influence functions approach [11]. Our results show that if the proportion of malignant neoplasms increased by 10%, the predicted Gini coefficient would increase by 7.2%; a 10% rise in heart disease would increase the Gini coefficient by 0.92%; and a 10% rise in cerebrovascular disease would increase the Gini coefficient by 1.5%. Serious diseases, such as malignant neoplasms and cardiovascular diseases, were prominent predictors of the skewness of health care expenditures. In just a few decades, the burden of disease in China has shifted considerably, with the epidemiological transition from acute diseases, such as infectious diseases, neonatal diseases, and childhood diseases to chronic diseases, such as cardiovascular diseases, tumors, degenerative diseases, and geriatric diseases [30]. Given the shift in China’s disease spectrum, our results predict a direct rise in the concentration of inpatient medical expenditures. Furthermore, the prevalence of chronic diseases, and trends of specific chronic diseases, has increased [29]. For example, cardiovascular hospitalization costs increased by more than 20% annually since 2004 [20], stroke prevalence increased by 155% and the incidence increased by 31.6% in rural areas from 1980s to 2013 [26]. China’s age-standardized incidence rates of cancer have continued to increase [4]. The prevalence and spending on tumors and cardiovascular diseases will continue to rise as China's demographics reflect population aging, prolonged life expectancy, increased expectation of medical care, and declining mortality rates, as well as the accumulation of risk factors.

Our study has limitations and strengths. First, we used administrative claims data at a prefecture-level city in central China, with the findings generalizable to developing central regions of China but not the whole country. Administrative claims data are not available for public use, and permission to access the unique administrative claims data meant the central regions, but not the city, could be identified. Second, variables not recorded in the administrative claims data, such as income and educational attainment, were not available. On the positive side, the patient-based administrative records avoid sample selection bias and information recall bias [9] and provide accurately recorded medical expenditure information and reliable clinical diagnosis, which infects survey studies of healthcare spending concentration [7]. Finally, our data provided the concentration of inpatient medical expenditures of patients covered by Urban Employee Basic Medical Insurance), but we did not address outpatient expenditures or the medical expenditures of other health insurance schemes, especially of the unemployed, students and children.

## Conclusions

Utilizing individual-based inpatient claims records, we provided abundant evidence on the characteristics and drivers of the concentration of inpatient medical expenditures in China. Medical expenditures exhibited an extreme skewed distribution, with a Gini coefficient of 0.588 and approximately 49% of annual inpatient medical expenditures generated by the top 10% of inpatient expenditures and the top 20% of inpatients accounting for 73.8% of inpatient expenditures. The predicted Gini coefficient would rise by 2.3% for an average increase of 1 day in the length of hospital stay, but the increase in tertiary care visits did not significantly increase concentration.

Our main contribution was to quantify the drivers of healthcare expenditure concentration. First, we found that HCUs tended to be elderly and male, with high frequency hospitalizations and long length of hospital stay. In terms of diseases profile, the top 10% of diagnoses were concentrated in diseases of the circulatory system, malignant neoplasms, diseases of the musculoskeletal system and connective tissue, diseases of the digestive system, injury, poisoning and certain other consequences of external causes, and diseases of the respiratory system. Among them, the incidence of ischemic heart disease, cerebrovascular diseases, and malignant neoplasms was high. We quantified the impact of the main disease drivers on healthcare expenditure concentration. With a 10% increase in the share of suffering from malignant neoplasms, the predicted Gini coefficient would increase by 7.2%; a 10% rise in heart disease would increase the Gini coefficient by 0.92%; and a 10% rise in cerebrovascular disease would increase the Gini coefficient by 1.5%. However, these significant positive effects on the concentration of inpatient expenditures were not observed in hypertension and diabetes. One reason might be that these diseases were mainly treated in outpatient health facilities.

Our study makes several contributions to the healthcare literature. First, with major shifts in China’s disease spectrum [30], our results show that changes in specific diseases will have a profound impact on the concentration of healthcare expenditures. To manage the increased HCU health expenditure concentration, healthcare administrators will need to invest in early detection and intervention management of specific chronic diseases and high-risk populations, especially the early diagnosis and treatment of key cancers. Finally, we extended and provided robust empirical evidence of the concentration of medical expenditures from a developing country.

## Data Availability

The data that support the findings of this study are available from Urban Employee Basic Medical Insurance but restrictions apply to the availability of these data, which were used under license for the current study, and so are not publicly available. Data are however available from the Corresponding author upon reasonable request and with permission of Urban Employee Basic Medical Insurance.
